# Supplementary material for: Computational drug repurposing strategy predicted peptide-based drugs that can potentially inhibit the interaction of SARS-CoV-2 spike protein with its target (humanACE2)
Source: PLoS One. 2021 Jan 8;16(1):e0245258. doi: 10.1371/journal.pone.0245258 (PMC7793299; doi:10.1371/journal.pone.0245258)
Supplement: S1 Table — (PDF) [file pone.0245258.s001.pdf]

Name	Structure of Structure	...	Poses	S score
Atosiban	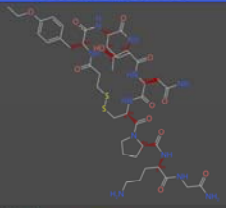	(	Pose 1	-9.9765167
Atosiban	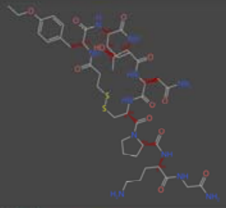	(	Pose 2	-8.3580637
Atosiban	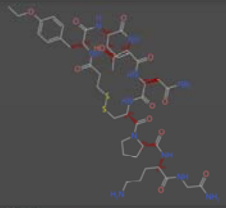	(	Pose 3	-8.0395441
Atosiban	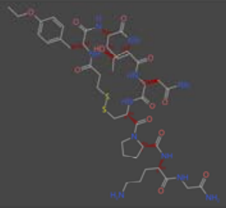	(	Pose 4	-7.7653613
Atosiban	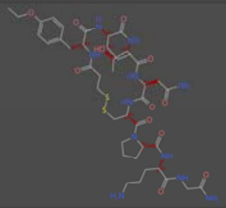	(	Pose 5	-7.6741271
BV2	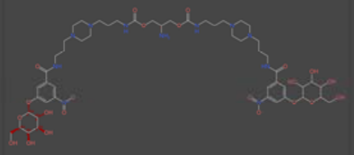	(	Pose 1	-10.552872
BV2	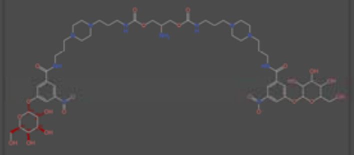	(	Pose 2	-9.9645472
BV2	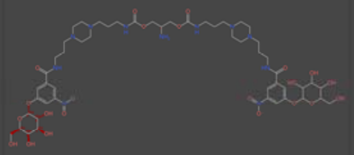	(	Pose 3	-9.7060776
BV2	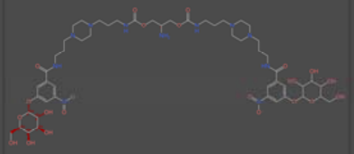	(	Pose 4	-9.6723614

Name	Structure of Structure	...	Poses	S score
BV2	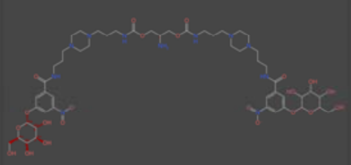	(	Pose 5	-9.6440516
Bleomycin	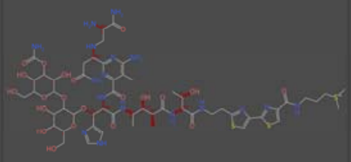	[:	Pose 1	-9.7330208
Bleomycin	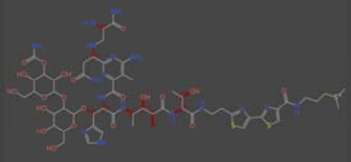	[:	Pose 2	-9.5062265
Bleomycin	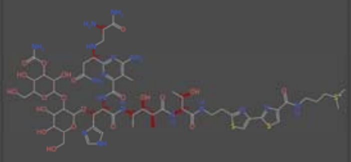	[:	Pose 3	-9.437006
Bleomycin	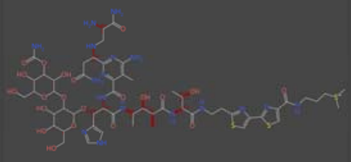	[:	Pose 4	-9.2959585
Bleomycin	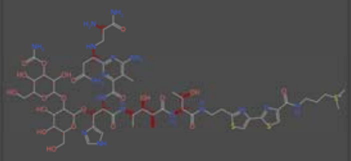	[:	Pose 5	-9.2261534
EXPT00686	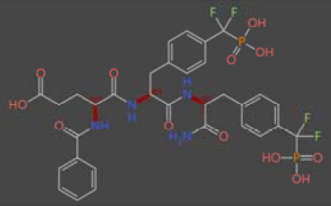	F	Pose 1	-7.6593571
EXPT00686	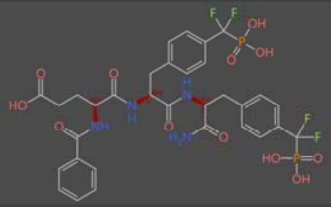	F	Pose 2	-7.5369987
EXPT00686	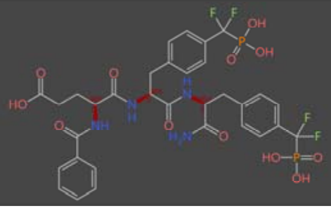	F	Pose 3	-7.4847755

Name	Structure of Structure	...	Poses	S score
EXPT00686	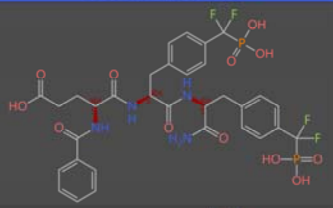	F	Pose 4	-7.3919034
EXPT00686	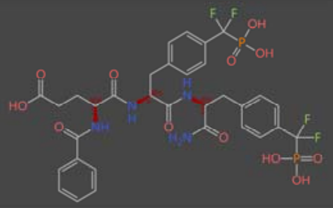	F	Pose 5	-7.3577948
EXPT00779	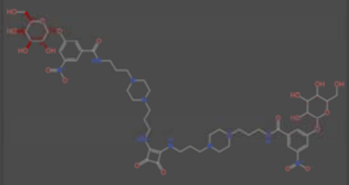	C	Pose 1	-10.334968
EXPT00779	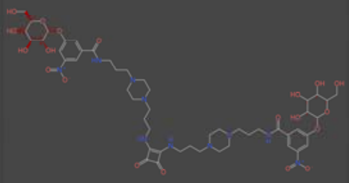	C	Pose 2	-9.976676
EXPT00779	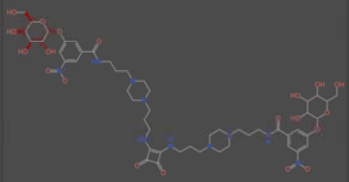	C	Pose 3	-9.7267323
EXPT00779	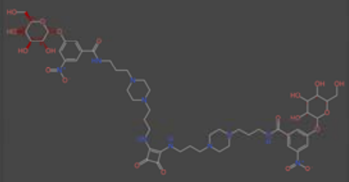	C	Pose 4	-9.5788488
EXPT00779	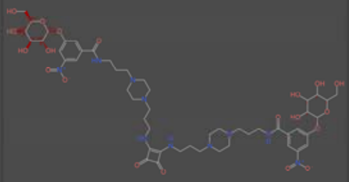	C	Pose 5	-9.3824739
Felypressin	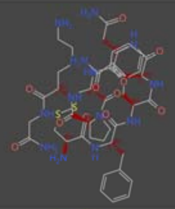	C	Pose 1	-8.3267183
Felypressin	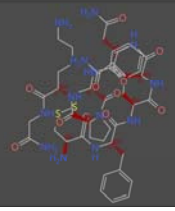	C	Pose 2	-8.2110882

Name	Structure of Structure	...	Poses	S score
Felypressin	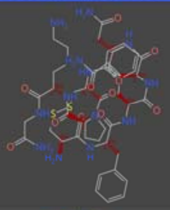	(	Pose 3	-7.9180994
Felypressin	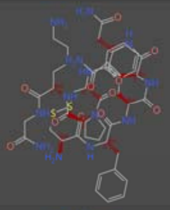	(	Pose 4	-7.7593579
Felypressin	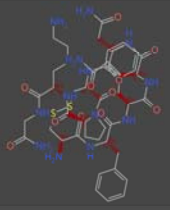	(	Pose 5	-7.3589697
Glycinamide Ribonucleotide	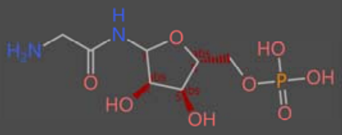	F	Pose 1	-5.5818815
Glycinamide Ribonucleotide	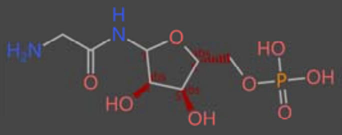	F	Pose 2	-5.3548274
Glycinamide Ribonucleotide	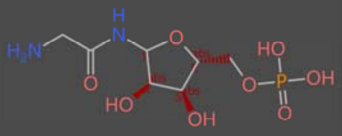	F	Pose 3	-5.2006378
Glycinamide Ribonucleotide	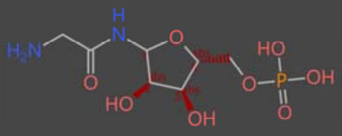	F	Pose 4	-5.1935925
Glycinamide Ribonucleotide	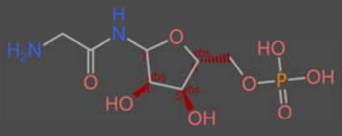	F	Pose 5	-5.130796
Gonadorelin	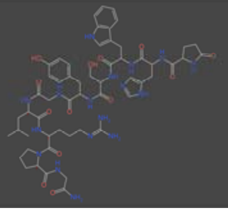	(	Pose 1	-9.8081579

Name	Structure of Structure	...	Poses	S score
Gonadorelin	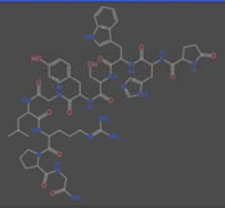	(	Pose 2	-9.7502747
Gonadorelin	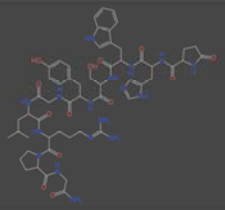	(	Pose 3	-9.4416246
Gonadorelin	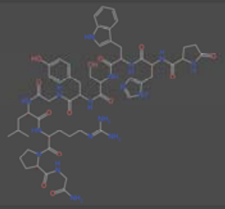	(	Pose 4	-9.4041004
Gonadorelin	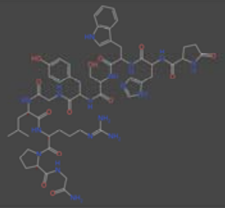	(	Pose 5	-9.3112593
Icatibant	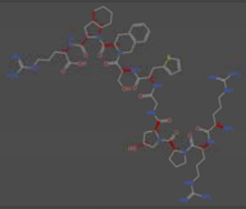	(	Pose 1	-9.6560278
Icatibant	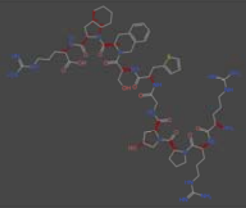	(	Pose 2	-9.6064682
Icatibant	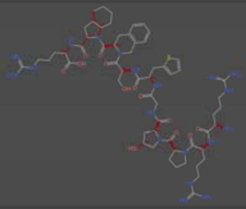	(	Pose 3	-9.5559416
Icatibant	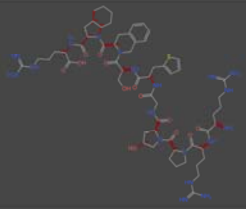	(	Pose 4	-9.4601755
Icatibant	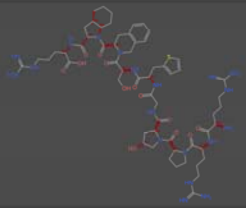	(	Pose 5	-9.1451349

Name	Structure of Structure	...	Poses	S score
Mixed Carbamic Phosphoric Acid Anhydride of 7 8-Diaminononanic Acid	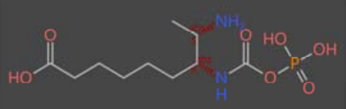	F	Pose 1	-5.7086005
Mixed Carbamic Phosphoric Acid Anhydride of 7 8-Diaminononanic Acid	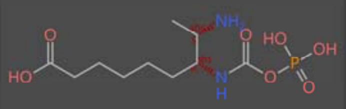	F	Pose 2	-5.7007484
Mixed Carbamic Phosphoric Acid Anhydride of 7 8-Diaminononanic Acid	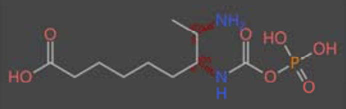	F	Pose 3	-5.5531178
Mixed Carbamic Phosphoric Acid Anhydride of 7 8-Diaminononanic Acid	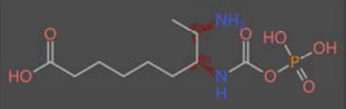	F	Pose 4	-5.4036312
Mixed Carbamic Phosphoric Acid Anhydride of 7 8-Diaminononanic Acid	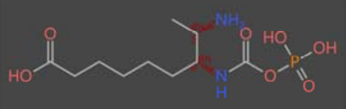	F	Pose 5	-5.4000859
Nafarelin	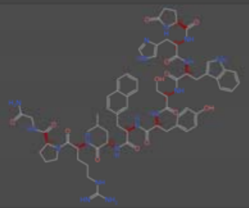	C	Pose 1	-9.7678156
Nafarelin	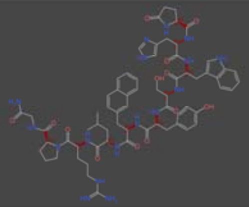	C	Pose 2	-9.5953712
Nafarelin	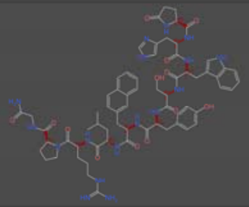	C	Pose 3	-9.5231056
Nafarelin	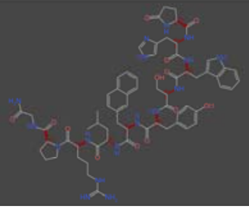	C	Pose 4	-9.3676577

Name	Structure of Structure	...	Poses	S score
Nafarelin	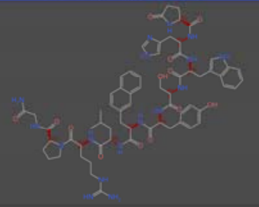	C	Pose 5	-9.1661434
P1-(adenosine-5'-P5-(uridine-5')pentaphosphate	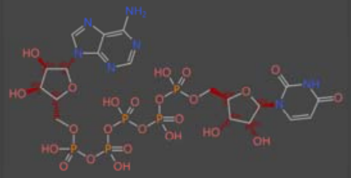	F	Pose 1	-9.0627356
P1-(adenosine-5'-P5-(uridine-5')pentaphosphate	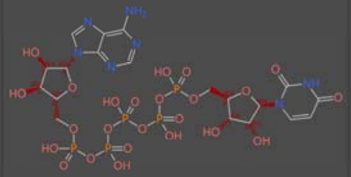	F	Pose 2	-8.5044508
P1-(adenosine-5'-P5-(uridine-5')pentaphosphate	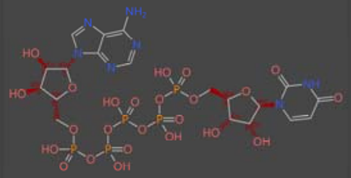	F	Pose 3	-8.4444618
P1-(adenosine-5'-P5-(uridine-5')pentaphosphate	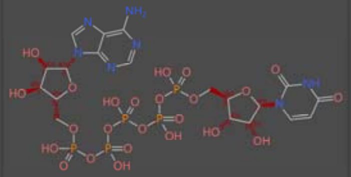	F	Pose 4	-8.3571167
P1-(adenosine-5'-P5-(uridine-5')pentaphosphate	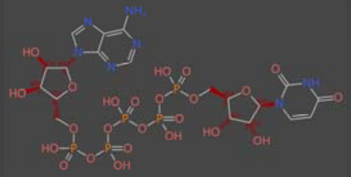	F	Pose 5	-8.232872
Sar9 Met (O2)11-Substance P	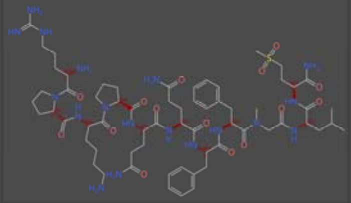	Ş	Pose 1	-10.627931
Sar9 Met (O2)11-Substance P	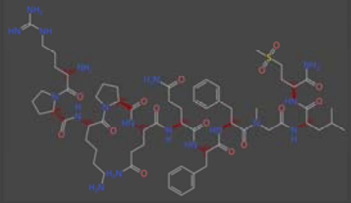	Ş	Pose 2	-10.590092
Sar9 Met (O2)11-Substance P	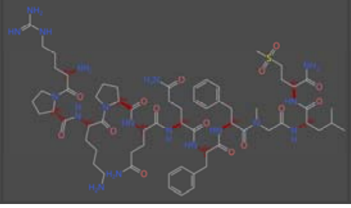	Ş	Pose 3	-10.522081

Name	Structure of Structure	...	Poses	S score
Sar9 Met (O2)11-Substance P	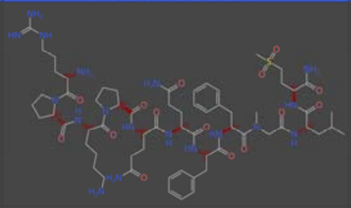	S	Pose 4	-10.442977
Sar9 Met (O2)11-Substance P	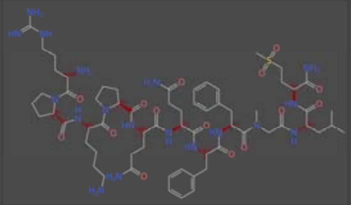	S	Pose 5	-10.328358
Serine-3'-aminoadenosine	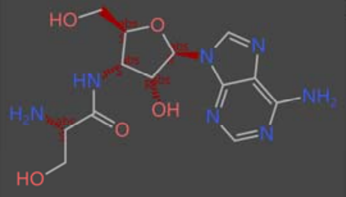	C	Pose 1	-5.8694892
Serine-3'-aminoadenosine	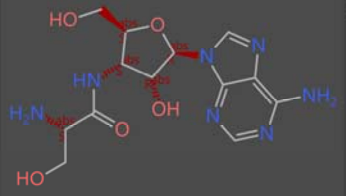	C	Pose 2	-5.8109426
Serine-3'-aminoadenosine	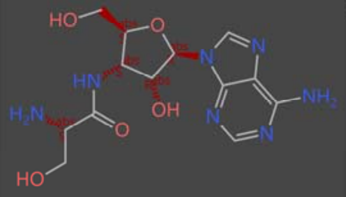	C	Pose 3	-5.7011609
Serine-3'-aminoadenosine	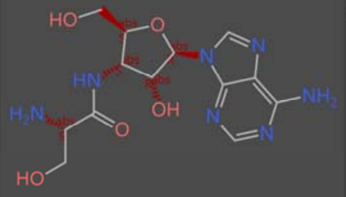	C	Pose 4	-5.6868701
Serine-3'-aminoadenosine	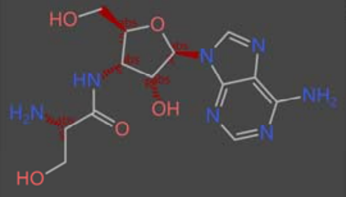	C	Pose 5	-5.6719384
Thymopentin	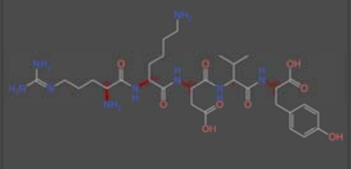	C	Pose 1	-7.547617
Thymopentin	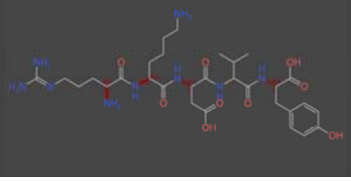	C	Pose 2	-7.3551712

Name	Structure of Structure	...	Poses	S score
Thymopentin	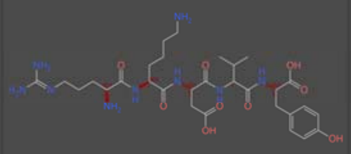	(	Pose 3	-7.2386456
Thymopentin	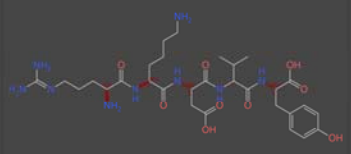	(	Pose 4	-6.8311429
Thymopentin	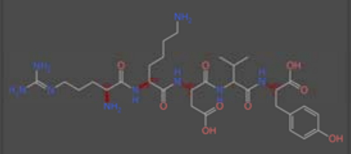	(	Pose 5	-6.7853174
p1-(5'-adenosyl)p5-(5'-thymidyl)pentaphosphate	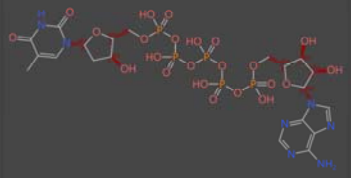	F	Pose 1	-8.6776609
p1-(5'-adenosyl)p5-(5'-thymidyl)pentaphosphate	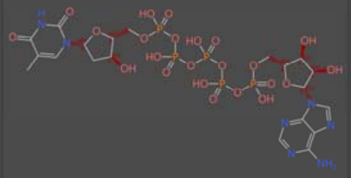	F	Pose 2	-8.5978622
p1-(5'-adenosyl)p5-(5'-thymidyl)pentaphosphate	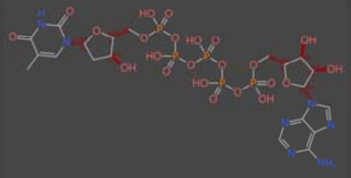	F	Pose 3	-8.2300749
p1-(5'-adenosyl)p5-(5'-thymidyl)pentaphosphate	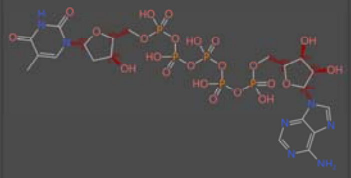	F	Pose 4	-8.2097178
p1-(5'-adenosyl)p5-(5'-thymidyl)pentaphosphate	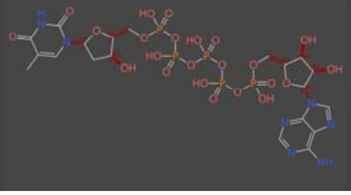	F	Pose 5	-8.1676407
