# Supplementary material for: Computational drug repurposing strategy predicted peptide-based drugs that can potentially inhibit the interaction of SARS-CoV-2 spike protein with its target (humanACE2)
Source: PLoS One. 2021 Jan 8;16(1):e0245258. doi: 10.1371/journal.pone.0245258 (PMC7793299; doi:10.1371/journal.pone.0245258)
Supplement: Attachment — Submitted filename: Egieyeh Response to Reviewers Letter.docx [file pone.0245258.s002.docx]

Dr Samuel A. Egieyeh

Pharmacology and Clinical Pharmacy

School of Pharmacy

University of the Western Cape

Bellville

7535

Cape Town

021 959 3388

12^th^ December 2020

## Editor-in-Chief

PLOS ONE

**Response Letter to the Reviewed Manuscript**

We appreciate the sincere and constructive review of the manuscript entitled “*Computational drug repurposing strategy predicted peptide-based drugs that can potentially inhibit the interaction of SARS-CoV-2 Spike proteins with its target (humanACE2)*”.

Following the review, which we accept in good faith, we have implemented the following changes according to the reviews (please note that number line of the previous version has changed in this new version):

The title has been slightly revised to reflect that the study was a computational prediction.

Reviewer #1: The aim of the study presented in this manuscript is to identify drugs from the Drug Bank that can prevent the binding of the SARS-CoV-2 spike protein with the human angiotensin-converting enzyme 2 (hACE2) receptor.

However, it is difficult to understand that the authors used the whole S(RBD)-ACE2 complex, instead of the binding site of the S(RBD) protein or ACE2 alone, for molecular docking. In general, the screened compound or peptide is supposed to competitively binding to the S protein or ACE2. It is also quite curious how the binding of candidate peptides with the S(RBD)-ACE2 complex can destroy the stability of the complex. Does it mean that huge conformational changes were induced by the binding of the candidate or there was great conflict inside the molecular docked conformation? The simulation duration (100 ps or 4 ns) is too short to generate reliable information or conclusion. The definition of RMSF is inappropriate: “The RMSF per residue is the distance between a residue in the first frame (structure) of the protein under simulation and the same residue in the subsequent frames (structures) of the same protein.” Most importantly, there is no any in vitro experiment to validate the modeling result.

1.	We revised the experiment and conducted the molecular docking on the hACE2 part of the S(RBD)-ACE2 complex. We have clarified that the molecular docking was done on the hACE2 (the spike protein interacting surface on hACE2) (Line 99 – 110)

2.	Simulation to get the average structure of the protein was done over a duration of 50 ns. (line 107)

3.	The definition of RMSF was revised (Line 156 – 158).

Reviewer #2: abstract - please change language to indicate all results are predictions, nothing was tested experimentally. for example: "The study identified a number of peptide-based drugs, including BV2, Gonadorelin,28 Icatibant, and Thymopentin that bind sufficiently" should be changed to "could potentially bind" or "is predicted to bind"

The statements were revised accordingly (Line 27 - 29).

Please clarify how many drugs were screened. The methods section said 7000, line 80 said 1101 and line 83 said 1070.

5.	It has been clarified in the document that the amount compounds docked in this study was 1070. These compounds were the top subset from the drug bank database (over 7000 compounds) virtually screened against the spike protein of Human *coronavirus HKU1* (HCoV-*HKU1*) in an inhouse study we conducted our lab (Line 92 – 94).

Line 165 - "These observations suggest potential conformational changes and moderate flexibility of the S protein and the hACE2 receptor complex in vivo" - I would suggest removing the in vivo from the sentence.

The word “in vivo” has been removed.

Figure 1 - please indicate which color is the structure from PDB and which is the average of the simulation.

Correction effected: Inserted is the three-dimensional (3D) superimposition of the average structure of the SARS-CoV-2 S protein and hACE2 complex generated from the molecular dynamics simulation (magenta) and the crystal structure of the SARS-CoV-2 S protein and hACE2 complex downloaded from RSCB protein data bank (cyan blue) (B). (Line 182 – 185)

Line 178 "3.2 Docking studies identified drugs with good binding affinities to the target site" - since binding affinities were not measured, this should be changed to something like "3.2 Docking studies identified drugs with potential to bind to the target site"

Corrected as suggested and rephrased (Line 188)

Lines 184-186: “The more negative the S score, the stronger the binding affinity, the better the ligand interaction with the protein and the more stable the complex of the drug hits with the protein complex.” Again, language should indicate this is all predicted and not experimentally tested.

It has been rephrased accordingly (Line 195 – 196)

Figure 4 – please indicate what the red and blue lines are in the figure itself for better readability (it appears in the text). For the text and legend of figure 4, please indicate the drug name (it is only listed as “the top hit”).

Corrected as directed. Legend inserted for the figure (now Figure 6) and top hit now specified (Line 305 – 314).

Line 253 – “Top-ranked drug hits identified in the study could inhibit protein-protein interaction between the SARS-CoV-2 spike protein and hACE2.” This sentence is misleading, as it suggests experimental evidence of inhibiting the binding. Please delete it.

Done. Deleted

Lines 261-263: “Similar hinderance to protein–protein interaction necessary for the establishment of the infection by coronavirus was observed for all of the top six ranked drug hits.” – please provide the data, either in the text or a supplementary file.

Statement has been deleted. Experiment was conducted on one predicted drug hit and presented as such.

Figure 5 – please indicate what the red and blue lines are in the figure itself for better readability (it appears in the text).

Done as suggested (see Figure 5).

Line 280 – “from our previous study”. Please provide a reference

Statement rephrased.

Lines 289-291: “The molecular docking studies identified drugs that bind strongly to the interacting surface of SARS-CoV-2 spike protein hACE2 complex.”. Please change to indicate these MAY bind strongly, as it is only predicted.

Statement rephrased to suggest that it was a prediction (340 – 342).

Lines 322-326: “Although a number of known drugs including hydroxychloroquine [5], Azithromycin [5], remdesivir [6], and idasanutlin [7] have been proposed and reported to be effective in the treatment of COVID-19, however results from our study suggest that hydroxychloroquine and remdesivir may have lower potential (as evidenced by binding free energy lower than -5.0 kcal/mol) to inhibit the interaction between the spike protein and hACE2.” There are several problems here. One – azithromycin and hydroxychloroquine have been widely shown to not be effective in treating COVID-19 patients, and should be removed from this sentence. Two – remdesivir is an RdRp inhibitor and is not expected to interfere with Spike binding to Ace2. Three- Ref #7 is a “thought experiment” is using idasanutlin to treat COVID-19, again in a mechanism unrelated to Spike-Ace2 binding. I suggest you delete these sentances.

Deleted as suggested.

In the Conclusion section (lines 327-337), please rephrase language to indicate potential inhibition rather than actual inhibition.

Done!

We believe that the changes made in this manuscript in line with the suggestions from the reviewers meet your expectations.

Thank you for your consideration of this manuscript for publication in your journal.

Sincerely,

Dr Samuel A. Egieyeh
